# Neurofilament Light Protein as a Biomarker in Severe Mental Disorders: A Systematic Review

**DOI:** 10.3390/ijms26010061

**Published:** 2024-12-25

**Authors:** Rosanna Squitti, Antonio Fiorenza, Alessandra Martinelli, Viviana Brembati, Daniela Crescenti, Mauro Rongioletti, Roberta Ghidoni

**Affiliations:** 1 Department of Laboratory Science, Research and Development Division, Ospedale Isola Tiberina—Gemelli Isola, 00186 Rome, Italy; maurociroantonio.rongioletti@fbf-isola.it; 2 Molecular Markers Laboratory, IRCCS Istituto Centro San Giovanni di Dio Fatebenefratelli, 25125 Brescia, Italy; vbrembati@fatebenefratelli.eu (V.B.); rghidoni@fatebenefratelli.eu (R.G.); 3 Department of Psychology, Uninettuno University, 00186 Rome, Italy; a.fiorenza2@students.uninettunouniversity.net; 4 Unit of Epidemiological and Evaluation Psychiatry, IRCCS Istituto Centro San Giovanni di Dio Fatebenefratelli, 25125 Brescia, Italy; amartinelli@fatebenefratelli.eu

**Keywords:** biomarker, bipolar disorder, blood, CSF, differential diagnosis, major depressive disorder, neurofilament light, psychiatric disorders, schizophrenia, severe mental disorders

## Abstract

Severe mental disorders (SMDs), such as schizophrenia (SZ), bipolar disorder (BD), and major depressive disorder (MDD), are heterogeneous psychiatric diseases that impose a significant societal burden due to their chronic disabling nature. There are no objective and reliable diagnostic tests for SMDs; thus, there is an urgent need for specific biomarkers to improve diagnosis, treatment, and resource allocation. Neurofilaments, found in cerebrospinal fluid and blood, offer reliable diagnostic and prognostic potential. This review discusses the link between neurofilament light chain (NfL) involvement in psychiatric and neurodegenerative diseases and gives insights into the diagnostic and prognostic value of NfL in SMDs. This systematic review searched PubMed, Scopus, and Web of Science databases to answer the research question “Are NfL levels higher in individuals with SMDs compared to healthy controls?” using terms related to neurofilament, SMDs, SZ, BD, and depression. Of 8577 initial papers, 115 were relevant. After exclusions and manual additions, 17 articles were included. Studies indicate elevated NfL levels in SMDs compared to healthy controls, suggesting its potential as a biomarker for SMDs and for distinguishing neurodegenerative diseases from psychiatric disorders. However, further longitudinal research is needed to confirm its reliability for differential diagnosis, disease prediction, and treatment assessment in psychiatry.

## 1. Introduction

Severe mental disorders (SMDs), including schizophrenia (SZ) and other psychotic conditions, bipolar disorder (BD), and major depressive disorder (MDD), represent a significant burden for society, mostly due to their chronically disabling nature [1]. They are classified according to predominant symptoms, their severity, and duration (DSM-5-TR). There is no objective and reliable diagnostic test for SMDs: the diagnosis is mostly based upon patients’ symptoms and caregivers’ reporting. Furthermore, their burden is likely to have been underestimated, and mental health organizations do not always provide adequate services and interventions for SMDs [2]. Hence, the need is urgent for true, unbiased, and specific markers to facilitate the diagnosis and prognosis of SMDs to improve the use of mental health organizations’ resources, the response to treatment, and outcomes for people with SMDs and their families [3,4].

Neurofilaments are a family of neuronal-specific intermediate filaments. They are thin and convoluted in dendrites and neuronal cell bodies, whereas they are abundant in axons, primarily in large myelinated ones, and determine the axonal caliber. Neurofilaments interact with several proteins, as well as cell organelles, including mitochondria [5]. As cytoskeletal components, neurofilaments are synthesized in the cell body and then transported distally along axons. In healthy neurons, normal neurofilament turnover, which is slow, and intracellular assembly are regulated by the ubiquitin–proteasomal system and, possibly, by the autophagy pathway [6,7]. The biological functions of neurofilaments are diverse and include supporting axonal stability and high-velocity nerve conduction; maintaining mitochondrial stability, the cytoskeletal content of microtubules [8], and the structure and function of dendritic spines; and regulating glutamatergic and dopaminergic neurotransmission at the synapse [9,10]. Different populations of neurofilament proteins at synapses, particularly at postsynaptic sites, differentially modulate neurotransmission, potentially influencing synaptic function in neuropsychiatric disorders [11]. Neurofilaments are classified into three subunits: heavy (NfH), medium (NfM), and light (NfL) chains. Among the neurofilament proteins, NfL is the most abundant. During processes of axonal damage caused by neurodegenerative, neuroinflammatory, traumatic, or vascular injuries, NfL is released into the brain interstitial fluid, which communicates freely with the cerebrospinal fluid (CSF) and the blood [5,12,13,14,15,16]. The exact mechanisms of NfL release from damaged or degenerating neurons are not fully understood, but active release through exosomes or passive release due to loss of integrity of the neuronal membrane may be involved [6]. Moreover, neurofilaments are released in CSF and blood during processes of axonal injury, encompassing aging, neuropsychiatric disorders, and neurodegenerative diseases [5].

NfL concentration in the blood is about 40-fold lower than in the CSF [17]. However, even in general circulation, NfL has been reported as a reliable surrogate biomarker of neuroaxonal injury in diverse neurological diseases, spanning from multiple sclerosis (MS) to Alzheimer’s disease (AD), frontotemporal lobar degeneration (FTLD), and traumatic brain injury [17,18,19,20,21]. Furthermore, mutations in the NF gene have been reported in multiple familial neurodegenerative disorders characterized by neurofilament aggregation. Failure of transport caused by NF mutations is thought to cause further accumulation of neurofilaments. This has been described for 14 mutations in the NF-L gene in the 2E and 1F forms of Charcot-Marie-Tooth disease [22].

SIMOA^®^ is a high-throughput Single-Molecule Array technology suitable for ultra-sensitive assays. It has been used for the detection of NfL in various biological matrices, showing high reliability in detecting NfL levels associated with neuroimaging abnormalities of white matter fibers, or gray matter changes such as global brain volume or local hippocampal areas under various conditions of brain structural changes [23,24,25].

The abnormal assembly of neurofilaments was found in several human neurodegenerative diseases and represents a potential diagnostic and prognostic test for several SMDs [3]. Even though the presence of alterations in brain structures in SMDs is still a debated issue [26,27], brain changes including white and gray matter structures in SZ have been reported [28], as well as abnormalities in white matter fibers and myelin sheets in MDD and BD [29,30]. NfL demonstrated high accuracy in longitudinally distinguishing between neurodegenerative and psychiatric disorders in patients with SMDs [31].

Herein, in this review, we report observations and opinions that attempt to structurally discuss the link between NfL involvement in SMDs in comparison with certain neurodegenerative diseases. In particular, we provide a brief overview of SMDs, the structure, function, and mechanisms of release of NfL, its use as a biomarker in neurodegenerative diseases, and its potential diagnostic and prognostic value in SMDs.

## 2. Methods

This systematic review was conducted in accordance with the 2020 updated Preferred Reporting Items for Systematic reviews and Meta-Analyses (PRISMA) recommendations [32].

### 2.1. Search Strategy

**Figure 1:**
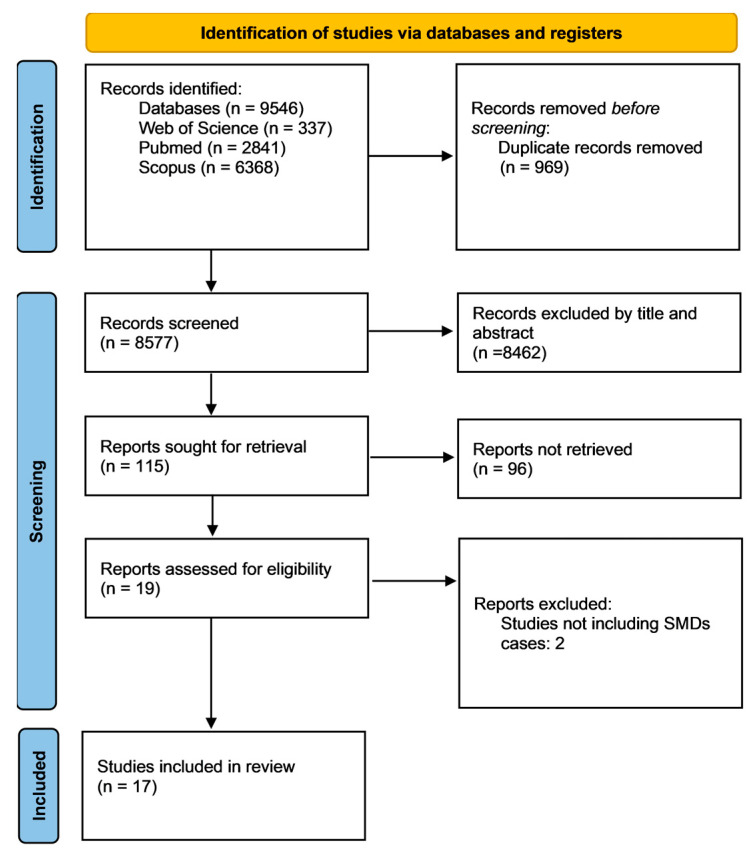
PRISMA flow diagram showing the inclusion and exclusion process of relevant studies. Abbreviations: n, numbers; SMDs, severe mental disorders.

The search strategy of this systematic review was designed with the research question, “Are NfL levels higher in individuals with SMDs compared to healthy controls?” From database establishment to 20 June 2024, two researchers (A.M. and A.F.) independently recovered eligible peer-reviewed literature on NfL levels in patients from different databases, namely PubMed, Scopus, and Web of Science. A search strategy was made for PubMed with truncations, Boolean operators, and Medical Subject Heading (MeSH) terms. This was peer-reviewed by a second researcher (A.F.) in accordance with the Peer Review of Electronic Search Strategies: 2015 Guideline Statement [33]. The searched keywords included “neurofilament”, “severe mental disorder”, “severe mental illness”, “schizophrenia”, “schizophrenia spectrum disorder”, “bipolar disorder”, “depression”, “major depressive disorder”, and their combinations. The manual exploration of citations in the relevant articles, alongside checking forward citations, was carried out. The PRISMA flow diagram was used to outline the process (identification, screening, and inclusion) of selecting relevant studies, as shown in Figure 1.

### 2.2. Inclusion and Exclusion Criteria

The inclusion criteria were as follows: controlled studies in which NfL levels were measured in patients (blood and CSF). The confirmed cases were considered regardless of age, ethnicity, and gender. The exclusion criteria were as follows: any irrelevant studies, abstracts, qualitative, case series, randomized controlled trials (RCTs), policy, case reports, reviews, opinion reports, communications, editorials, animal studies, in vitro studies, and articles without available full texts were excluded. Two researchers (A.M. and A.F.) autonomously finalized this process, and any differences were resolved by the third investigator (R.S.).

### 2.3. Data Extraction

The following data were extracted from the eligible studies: first author, year of publication, study design, method for determination of NfL levels, number of participants, NfL levels in patients and control subjects, and other important information related to the studies. Serum/plasma/whole blood/CSF NfL concentration units were recalculated to pg/mL. These baseline characteristics of included studies were tabulated independently by two investigators (A.M. and A.F.), and any disagreements were settled via consensus.

## 3. Results and Discussion

Of 8577 initial papers; 115 were relevant. After exclusions and manual additions, 17 articles were included (Figure 1; Table 1). Table 1 shows the extracted data from included studies, and sex concordance (percentage male/female) between the case sample and healthy control (HC) groups in the different studies.

### 3.1. NfL and SMDs

#### 3.1.1. The Brain Abnormalities

Although the presence of brain alterations that occur after the onset of the first psychotic symptoms is still an unresolved question [26,27], structural brain alterations in SZ have been reliably observed [28]. A recent study observed increased NfL levels in children and adolescents with SZ and BD, suggesting neuronal damage may play a role in their pathophysiology and may be more pronounced in SZ [50]. However, other studies reported no elevation of plasma NfL in individuals experiencing first-episode psychosis, in those at ultra-high risk for psychosis [31], or in untreated psychosis [51]. In a study by Cilia and colleagues [28], bilateral insula thinning linked with elevated plasma NfL was found in treatment-resistant SZ, indicating a neurodegenerative process [34].

A decrease in myelin in the high caliber axons of the callosal splenium was observed in brain specimens of patients affected by MDD, but not in those with SZ [29]. Similarly, in MDD, mixed patterns of structural brain abnormalities have been reported in patients with recurrent depressive episodes [52,53]. Nevertheless, the biological processes underlying these structural abnormalities remain obscure to date. The association of negative symptom severity with altered white matter long fibers in SZ [54,55,56], as well as the association of depressive symptoms with brain structural abnormalities (e.g., in white matter, hippocampal, and frontal structures) in MDD [35,57], support the involvement of axonal injury in SMDs. Furthermore, it has been reported that the morphological findings in primary psychiatric disorders may be associated with the decline of cognitive function observed in the patients [58,59,60,61]. This prompted the investigation of neurofilaments as dynamic biomarkers of damage in myelinated axons or alteration of axonal stability in high-velocity conduction nerves [22] in neuropathological active processes of SMDs.

Among the first studies, Jakobsson and colleagues [36] showed increased NfL concentrations in the CSF of well-characterized BD patients compared to healthy controls (Table 1). The authors defined the abnormalities observed in the patients as ongoing lesions that resemble those of small vessel disease because they noted that many of the affected cognitive domains and anatomical abnormalities in BD resembled the symptoms of small-vessel disease. Interestingly, treatment with atypical antipsychotics, including olanzapine and quetiapine, appeared to be associated with increased NfL levels [36]. Nevertheless, a subsequent prospective study investigating CSF markers of neuroinflammation and neuronal injury did not show evidence that NfL could predict clinical outcomes in a long-term follow-up of patients with BD [62]. A recent study reported individuals with BD and healthy controls with no significant differences in NfL levels except for those with a disorder duration exceeding 3 years [63].

Several studies reported increased values of NfL in major affective disorders but not in SZ [23,37]. A recent study reported that higher NfL levels were linked to reduced brain volumes, particularly in younger-onset dementia patients, but not in those with MDD or SZ [64]. Investigating the underlying factors of late-life depression, Gudmundsson and colleagues demonstrated that women affected by geriatric depression showed higher CSF NfL levels compared with women without depression [38]. In addition, a study involving 3895 participants found that higher levels of plasma NfL are associated with more depressive symptoms cross-sectionally and an increased risk of incident depressive events longitudinally, indicating a potential role of neuroaxonal damage in the development of late-life depression [65].

Of note is the association between plasma levels of NfL and white matter microstructure integrity in BD [39]: the evidence shows that NfL levels are increased in BD and might be related to plasticity processes of neuronal connectivity remodeling [39]. NfL was shown to be higher and associated with a greater deficit in executive function in MDD [37] and associated with aging in BD [66]. Moreover, elevated serum NfL in suicide attempters diagnosed with BD and MDD [40] is consistent with neuroimaging studies showing periventricular white-matter hyperintensities and neuroaxonal damage associated with suicidal behavior [67]. Although NfL serum levels were significantly higher in attempted suicide patients compared to healthy controls, a significant association between NfL levels and risk factors for suicide was not found [40].

However, results on the association between NfL and psychiatric disorders were not univocal: Besse et al. demonstrated that NfL concentrations did not change between patients with MDD and healthy controls [68]. In the same line, Al Shweiki and colleagues [41] showed no difference from healthy controls in BD, SZ, and MMD, even though they compared the small size groups of SMDs patients. Interestingly, with respect to these SMDs, values of NfL were increased in the serum of FTLD. Thus, the authors proposed NfL as a reliable biomarker for differential diagnosis. These results were confirmed by Katisko [42], showing in a bigger patient sample the utility of NfL concentrations as a potential biomarker for differential diagnosis. Specifically, an NfL cut-off of 20 pg/mL highly specifically differentiated FTLD from SMDs, namely patients with psychotic and mood disorders [42,69] (Table 1). The higher the serum NfL concentrations, the lower the Mini-Mental State Examination (MMSE) and Activities of Daily Living for Mild Cognitive Impairment (ADCS-ADL) outcomes in primary psychiatry disorders plus FTLD [42]. Furthermore, plasma NfL values were associated with greater deficits in executive function in MDD [37].

#### 3.1.2. The Symptom Dimension

Some consistent findings linked the alteration of long association white matter fibers in SZ to negative symptoms and deficits in several cognitive domains [54,55,56].

Biomarker-driven approaches were proposed to guide the identification of SZ subtypes [70,71]. However, given the dynamic nature of the NfL response, the high variance evidenced in SZ may reflect different clinical stages, according to the non-linear individual trajectories of white matter integrity on the course of the disorder [72]. Moreover, a clozapine-treated subgroup of SZ reported an increase of NfL [73], while treatment-resistant SZ does not appear to be associated with axonal degeneration [74].

In BD, NfL levels increase with age [39,68]. A higher concentration of NfL was associated with decreased cognitive performance [43]. High NfL may reflect subtle neurodevelopmental deficits that may be a trait risk factor for BD or an indicator of brain injuries that will manifest later in life as cognitive impairments and/or disease progression [36]. No associations between NfL and subtypes of BD diagnosis were described, suggesting that NfL is not a useful diagnostic marker of BD subtypes. However, high NfL is related to a certain subtype of bipolar patients with lower global functioning and more likely to be treated with atypical antipsychotics because of a more severe disorder [36]. A case–control study reported weak associations between NfL and cognitive domains in BD, none of which remained significant after adjusting for multiple testing, leaving it uncertain whether this association is a general phenomenon or specific to BD [44].

In samples of patients with MDD, some studies associated structural brain abnormalities mostly with depressive symptoms and cognitive dysfunctions [35,57]. While preliminary cross-sectional and case–control studies have yielded inconsistent results regarding the association between NfL and symptomatology [75], more consistent results have been described for cognition. A negative association was described between cognitive processing speed and NfL levels in MDD [23,76]. An exploratory cross-sectional study found that, after adjusting for potential confounders, NfL levels, depressive symptoms, and cognitive decline are interconnected, with NfL levels potentially mediating the relationship between depressive symptoms and cognitive decline in older adults [77]. As cognitive processing speed was predicted by both age and NfL levels and it was already recorded to negatively correlate with NfL levels in elderly individuals and patients with neurodegenerative diseases [18,78], the decline of processing speed in patients with MDD should be seen as an interaction of physiological (age-related) and pathological (disorder-related) degenerative brain process [23]. In MDD patients over 65 years of age, higher plasma NfL levels are negatively correlated with cognitive performance, particularly in those clustered with neurodegenerative disorders like AD [45]. These results suggest that NfL could be a useful biomarker for predicting cognitive decline in MDD patients. In MDD, high NfL levels were also related to executive dysfunction [37]. Considering different depression subtypes, a correlation between high NfL levels and treatment resistance in major depression was reported [79], and contrasting results were described in first-episode and medication-naïve patients with MDD [35].

#### 3.1.3. NfL, SMDs, and Other Elements

A study found an interaction between high vitamin D deficiency and NfL in BD, affecting cognitive symptoms with an impact on neurocognitive scores and verbal fluency across all ages and on processing speed in younger individuals [80].

A study by Huang et al. (2023) found that individuals with ketamine dependence and MDD had significantly higher NfL levels, suggesting increased neuroaxonal pathology due to the interaction between ketamine dependence and MDD, rather than MDD alone [81]. Therefore, baseline NfL concentrations may predict the antidepressant effects of low-dose ketamine infusion in treatment-resistant MDD [46].

Another study found elevated levels of NfL and Procollagen type 1 N-terminal propeptide (P1NP), a bone turnover biomarker, in BD and MDD, indicating a dysregulated brain–bone axis, cognitive impairment, and systemic inflammation [82].

Results in serum levels of NfL and glial fibrillary acidic protein (GFAP) in MDD are controversial [83,84]. Steinacker and colleagues investigated the utility of serum GFAP for the differential diagnosis and monitoring of MDD and found the highest GFAP levels in MDD compared to healthy controls, SZ, and BD, while NfL serum levels were not different between groups and not associated with GFAP levels [47].

Another study suggested that NfL could be associated with neuroimmune activation (specifically with the 18 kDa translocator protein TSPO) but not clearly identifiable in an early, mostly untreated psychosis sample, including those at high risk [51].

### 3.2. Differential Diagnosis Between SMDs and Neurodegenerative Diseases Using NfL

Elevated NfL levels are consistently reported in neurodegenerative diseases such as AD, MS, and amyotrophic lateral sclerosis (ALS), along with other neurological conditions characterized by neuroaxonal damage [14,15,17,18,19,20,21,85,86]. In addition, NfL was also reported to be significantly higher in neurodegenerative or neurological disorders than in SMDs. Thus, NfL shows promise as a diagnostic test to assist with the often-challenging diagnostic dilemma of distinguishing SMDs from neurodegenerative and neurological disorders [3,42,87,88]. In particular, differences were found between SMDs and FTD [42], especially in individuals aged 60 or younger, although its accuracy diminished with increasing age [89].

Using large reference cohorts, Eratne and colleagues found a high accuracy of NfL in distinguishing behavioral variant frontotemporal dementia (bvFTD) from MDD, BD, and treatment-resistant SZ by comparing plasma NfL levels across various conditions [90]. On the contrary, another study showed no significant differences between participants with and without a history of MDD in terms of AD biomarkers, white matter hyperintensity (WMH) burden, NfL, cognitive scores, age of symptom onset, disease duration, or vascular risk scores [91]. Interestingly, a recent study concluded that urine is not a suitable matrix for analyzing NfL to distinguish FTD from SMDs [92].

Elevated plasma NfL levels correlate with increased depressive symptoms in late life, suggesting that neuroaxonal damage may play a pivotal role in late-life depression, potentially surpassing associations seen with other biomarkers such as total tau and β-amyloid [65].

### 3.3. Discussion

SMDs affect more than 4% of the general population and are usually long-lasting [1].

As per the 5th Edition of the Diagnostic and Statistical Manual of Mental Disorders and its revision [93], SZ is typified by continuous or relapsing psychotic episodes, including hallucinations, delusions, paranoia, and decreased emotional expression [94]. SZ also distorts thoughts and perceptions and causes social withdrawal.

BD and MDD are mood disorders characterized by significant changes in mood state and have a high prevalence in the general population. BD disturbs around 1% of the global population [95], while MDD affects 10.4% of the global population with a lifetime prevalence of up to 20.6% [96].

SMDs are frequently associated with other mental disorders, including substance abuse, anxiety disorders, and suicidal behaviors [97]. SMDs highly impact healthcare providers and society, representing global health problems since they impose a significant burden on patients and caregivers due to their chronic disabling and recurring nature. The World Health Organization reports that only MDD accounts for the utmost burden related to nonfatal health outcomes, accounting for 34.1 million total years lived with disability [98]. SMDs are complex diseases typified by heterogeneous etiology, spanning from genetic predisposition to environmental factors. Epigenetics further complicates the understanding of the underlying biological mechanisms.

Even though a certain number of studies have a higher percentage of women (Table 1), most studies report a concordance in the sex distribution (percentage male/female) between the case sample and HC groups in the different studies. Despite the lack of objective and reliable diagnostic tests for SMDs, high-throughput technologies have recently become available, enabling new approaches and research to bridge the gap between the lack of biological insights into SMDs and disease pathogenesis, as well as therapeutic outcomes. In the last decade, SIMOA^®^ technology promoted a widespread application of NfL ultra-sensitive assays in serum/plasma [99].

#### 3.3.1. Role of High-Throughput Technologies, Challenges, and the Need for Longitudinal Studies

High-throughput technologies like SIMOA^®^ have revolutionized the study of NfL in psychiatric disorders [23,42,45], providing insights into disease mechanisms [39,67]. Although NfL alterations are not unique to psychiatric disorders and elevated NfL levels are a recognized biomarker of neuronal damage in different neurological diseases [14,17,85,86], NfL shows promise as a biomarker for neurodegenerative diseases and psychiatric conditions, particularly for the differential diagnosis between primary psychiatric disorders and FTD. However, the clinical utility of NfL in psychiatric disorders requires further validation through longitudinal studies [3,42,87,100]. These studies are essential to establish NfL as a reliable biomarker for differential diagnosis, disease monitoring, and the prediction of treatment response across various psychiatric conditions.

#### 3.3.2. Intersection of Neurobiology and Psychiatric Symptomatology

NfL levels have been associated with cognitive deficits [23,42,43,45,77,80], neurodegenerative changes [39], and treatment responses in SMDs [46]. Understanding these associations can enhance our knowledge of disease pathophysiology and potentially improve diagnostic and therapeutic strategies in psychiatry.

#### 3.3.3. Clinical Implications and Future Directions

In the following studies reported in Table 1 [3,35,36,37,38,39,40,43,44,46,48], NfL levels were higher in individuals with SMDs compared to HC. In the studies by Cilia B. et al., 2024, [34] and Knorr U. et al., 2022 [49], NfL levels are comparable between patients and HC, while in the study by Al Shweiki M.R. et al., 2019 [41], NfL levels are comparable between patients with depression and HC, higher in BD patients compared to HC, and lower in SZ patients compared to HC. In the study by Steinacker P. et al., 2021 [47], Nfl levels are higher in individuals with SMDs (BD and MDD) compared to HC and comparable in individuals with SZ compared to HC. Elevated NfL levels in these psychiatric disorders suggest potential applications in clinical practice for early diagnosis, the monitoring of disease progression, and the assessment of treatment efficacy [46,73,100]. While the lack of specificity and elevated levels of NfL in both psychiatric and neurological disorders may limit the clinical utility of NfL as a pathology biomarker for SMDs, it may find application as an aid in the diagnostic assessment of psychiatric disorders. Indeed, recent studies have shown that NfL may differentiate psychiatric disorders from neurological disorders, especially from bvFTD [3,41,42,87], which show significant symptomatic overlap and are commonly misdiagnosed in primary psychiatric disorders [88]. Continued research is crucial to fully leverage NfL as a dynamic biomarker in psychiatric care, addressing current diagnostic challenges and improving patient outcomes.

#### 3.3.4. Strengths and Limitations

This systematic review offered a comprehensive synthesis of evidence on the topic of NfL in SMDs, minimizing bias and providing a consolidated view of findings from multiple studies. However, the reliability hinges on the quality of the included studies, which were heterogeneous.

## 4. Conclusions

Most of the studies on NfL investigation in SMDs show an increase in NfL concentrations in CSF and serum/plasma values in comparison with HC, even though these values are significantly lower than those found in neurodegenerative diseases [36,37,42]. Some authors have argued about the existence of a gradient of NfL alterations starting from cognitive normality to neurodegeneration, particularly FTLD, in which primary psychiatric pathologies may represent an intermediate state [3,42,87,88,100]. This gradient may reflect the state of alterations in the white matter fibers that are damaged in these disorders [23,24,25,35,39,72]. On this basis, peripheral NfL can be an emerging biomarker with potential in depression and cognitive function [45,77,100], and NfL detection can provide some utility for the differential diagnosis of neurodegenerative diseases, such as FTD, from psychiatric disorders [42].

These are encouraging results that may spread the application of high-throughput technologies in clinical applications for the differential diagnosis of mental disorders. The usefulness is immediately appreciable since the FTLD spectrum and SMDs have a significant clinical and symptomatology overlap, while they substantially differ in terms of the underlying pathophysiology and prognosis [42,87]. Understanding these associations can enhance our knowledge of disease pathophysiology and potentially improve diagnostic and therapeutic strategies in psychiatry. Elevated NfL levels in certain psychiatric disorders suggest potential applications in clinical practice for early diagnosis, the monitoring of disease progression, and the assessment of treatment efficacy [46,73,100]. Continued research is crucial to fully leverage NfL as a dynamic biomarker in psychiatric care, addressing current diagnostic challenges and improving patient outcomes. Nevertheless, the longitudinal prospective studies in the field are still scanty, and more research is required to assess NfL detection as a reliable biomarker for the differential diagnosis of mental disorders, to predict their onset and progression, to characterize the disorder, and to measure the effectiveness of potential psychiatric interventions and medications.

## Figures and Tables

**Table 1:** Summary of studies included after systematic review.

References	Control Samples, n	Age (SD) [IQR]	Sex (Percentage%) M; F	Blood NfL, pg/mL (SD) [IQR]	CSF NfL, pg/mL (SD) [IQR] {SE}	Case Samples, n (Disease)	Age (SD) [IQR]	Sex (Percentage%) M; F	Blood NfL, pg/mL (SD) [IQR]	CSF NfL, pg/mL (SD) [IQR] {SE}	Patients’ Conditions	Sex Concordance Between Case Samples and HC
[3] Eratne D. et al., 2020	21	66.00 [65.00–67.00]	5 (23.8); 16 (76.2)	n.a.	1036.00 [908.00–1165.00] ELISA	77 (NND)	57.00 [55.00–59.00]	49 (63.6); 28 (36.4)	n.a.	3560.00 [2918.00–4601.00] ELISA	NND, PSY, HC	No
						31 (PSY)	51.00 [47.00–55.00]	19 (61.3); 12 (38.7)		949.00 [830.00–1108.00] ELISA		No
[34] Cilia B. et al., 2024	43	39.60 (11.40)	27 (62.8); 16 (37.2)	6.10 [5.30–6.80] SIMOA^®^	n.a.	39	37.90 (8.40)	31 (79.5); 8 (20.5)	5.50 [4.60–6.40] SIMOA^®^	n.a.	TRS, HC	Yes
[35] Jiang L. et al., 2021	72	34.00 (12.00)	37 (51.4); 35 (48.6)	143.50 [73.60–339.30] ELISA	n.a.	82	34.00 (11.00)	32 (39.0); 50 (61.0)	405.80 [281.50–625.50] ELISA	n.a.	MDD, HC	No
[36] Jakobsson J. et al., 2014	86	35.00 [28.00–46.00]	39 (45.3); 47 (54.7)	n.a.	359.00 {34.00} ELISA	133	35.00 [28.00–50.00]	52 (39.1); 81 (60.9)	n.a.	480.00 {25} ELISA	BD, HC	Yes
[37] Chen M. et al., 2022	40	28.25 (14.08)	13 (32.5); 27 (67.5)	16.65 (8.07) ELISA	n.a.	40	28.25 (14.35)	13 (32.5); 27 (67.5)	28.76 (22.53) ELISA	n.a.	MDD, HC	Yes
[38] Gudmundsson P. et al., 2010	13	n.a.	0 (0.0); 13 (100.0)	n.a.	277.00 (186.00) ELISA	11	n.a.	0 (0.0); 11 (100.0)	n.a.	427.00 (318.00) ELISA	MDD, no DP as control	Yes
[39] Aggio V. et al., 2022	29	41.72 (10.19)	13 (44.8); 16 (55.2)	4.28 (2.39) SIMOA^®^	n.a.	45	48.20 (11.87)	10 (22.2); 35 (77.8)	9.13 (4.78) SIMOA^®^	n.a.	BD, HC	Yes
[40] Ramezani M. et al., 2022	35	30.00 (6.27)	10 (28.6); 25 (71.4)	13,730.00 (5,110.00) ELISA	n.a.	50 (22 MDD + 28 BD)	28.92 (11.34)	11 (22.0); 39 (78.0)	40,520.00 (33,540.00) ELISA	n.a.	MDD and BD with suicide attempts, HC	Yes
[41] Al Shweiki M.R. et al., 2019	27	46.80 [39.10–54.10]	10 (37.0); 17 (63.0)	15.10 [11.30–19.10] SIMOA^®^	n.a.	28 (DP)	52.10 [46.20–58.40]	13 (46.4); 15 (53.6)	15.70 [12.40–25.00] SIMOA^®^	n.a.	DP, BD, SZ, bvFTD, HC	Yes
						11 (BD)	51.40 [33.50–58.10]	7 (63.6); 4 (36.4)	17.80 [12.60–23.10] SIMOA^®^			No
						11 (SZ)	41.10 [31.40–48.50]	5 (45.5); 6 (54.5)	11.60 [9.80–23.50] SIMOA^®^			Yes
						20 (bvFTD)	50.60 [44.90–52.50]	10 (50.0); 10 (50.0)	72.70 [28.30–90.00] SIMOA^®^			No
[42] Katisko K. et al., 2020	34	55.70 (9.40)	14 (41.2); 20 (58.8)	15.50 (9.50) SIMOA^®^	n.a.	91	65.00 (8.70)	44 (48.4); 47 (51.6)	43.70 (36.30) SIMOA^®^	n.a.	FTLD, PPD as control	Yes
[43] Rolstad S. et al., 2015	71	37.80 (14.60)	27 (38.0); 44 (62.0)	n.a.	254.38 (55.42) ELISA	82	38.30 (12.50)	34 (41.5); 48 (58.5)	n.a.	485.73 (425.62) ELISA	BD, HC	Yes
[44] Knorr U. et al., 2024	44	30.50 [24.00–40.50]	25 (56.8); 19 (43.2)	5.73 [4.50–7.84] SIMOA^®^	354.50 [214.75–566.75] ELISA	85	33.00 [26.00–42.00]	44 (51.8); 41 (48.2)	6.87 [4.98–9.11] SIMOA^®^	336.50 [246.50–490.50] ELISA	BD, HC	Yes
[45] Chen C.Y. et al., 2024	17	77.12 (8.75)	3 (17.6); 14 (82.4)	26.21 (16.59) SIMOA^®^	n.a.	37	70.05 (7.10)	8 (21.6); 29 (78.4)	16.95 (10.19) SIMOA^®^	n.a.	MDD, MCI/AD	Yes
[46] Lin W. et al., 2023	17	43.94 (9.67)	8 (47.1); 9 (52.9)	22.72 (8.07) ELISA	n.a.	24	48.63 (8.12)	9 (37.5); 15 (62.5)	47.92 (21.23) ELISA	n.a.	TRD saline-treated, HC	Yes
[47] Steinacker P. et al., 2021	16	45.00 [27.00–64.00]	4 (25.0); 12 (75.0)	15.20 (7.10) SIMOA^®^	n.a.	45 (MDD)	48.00 [19.00–69.00]	16 (35.6); 29 (64.4)	29.30 (35.30) SIMOA^®^	n.a.	MDD, SZ, BD, HC	Yes
						9 (SZ)	33.00 [23.00–56.00]	4 (44.4); 5 (55.6)	15.60 (8.10) SIMOA^®^			Yes
						11 (BD)	48.00 [18.00–56.00]	8 (72.7); 3 (27.3)	21.20 (16.60) SIMOA^®^			No
[48] Al-Hakeim H.K. et al., 2023	47	38.00 (7.90)	22 (46.8); 25 (53.2)	11.10 (1.19) ELISA	n.a.	53	37.20 (11.10)	19 (35.8); 34 (64.2)	23.00 (1.13) ELISA	n.a	MDD, HC	Yes
[49] Knorr U. et al., 2022	44	30.00 [25.00–42.00]	(47.0); (53.0)	5.73 [4.50–7.84] SIMOA^®^	354.00 [214.00–566.00] ELISA	85	33.00 [26.00–42.00]	(50.0); (50.0)	6.81 [4.97–9.07] SIMOA^®^	332.00 [246.00–479.00] ELISA	BD, HC	No

Abbreviations: AD, Alzheimer’s disease; BD, bipolar disorder; bvFTD, behavioral variant frontotemporal dementia; CSF, cerebrospinal fluid; DP, depression; ELISA, enzyme-linked immunosorbent assay; F, female; FTLD, frontotemporal lobar degeneration; HC, healthy controls; IQR, interquartile range; M, male; MCI, mild cognitive impairment; MDD, major depressive disorder; n, number of samples; n.a., not applicable; NfL, neurofilament light; NND, neurodegenerative or neurological disorder; PPD, primary psychiatric disorders; PSY, psychiatric disorder; SD, standard deviation; SE, standard error; SIMOA^®^, Single-Molecule Array; SZ, schizophrenia; TRD, treatment-resistant depression; TRS, treatment-resistant schizophrenia. Control samples refer to healthy controls unless otherwise specified.
